# Binding Affinity via Docking: Fact and Fiction

**DOI:** 10.3390/molecules23081899

**Published:** 2018-07-30

**Authors:** Tatu Pantsar, Antti Poso

**Affiliations:** 1 School of Pharmacy, University of Eastern Finland, P.O. BOX 1627, 70211 Kuopio, Finland; tatu.pantsar@uef.fi; 2 Department of Internal Medicine VIII, University Hospital Tübingen, Otfried-Müller-Strasse 14, 72076 Tübingen, Germany

**Keywords:** docking, solvent effect, binding affinity, scoring function, molecular dynamics

## Abstract

In 1982, Kuntz et al. published an article with the title “A Geometric Approach to Macromolecule-Ligand Interactions”, where they described a method “to explore geometrically feasible alignment of ligands and receptors of known structure”. Since then, small molecule docking has been employed as a fast way to estimate the binding pose of a given compound within a specific target protein and also to predict binding affinity. Remarkably, the first docking method suggested by Kuntz and colleagues aimed to predict binding poses but very little was specified about binding affinity. This raises the question as to whether docking is the right tool to estimate binding affinity. The short answer is no, and this has been concluded in several comprehensive analyses. However, in this opinion paper we discuss several critical aspects that need to be reconsidered before a reliable binding affinity prediction through docking is realistic. These are not the only issues that need to be considered, but they are perhaps the most critical ones. We also consider that in spite of the huge efforts to enhance scoring functions, the accuracy of binding affinity predictions is perhaps only as good as it was 10–20 years ago. There are several underlying reasons for this poor performance and these are analyzed. In particular, we focus on the role of the solvent (water), the poor description of H-bonding and the lack of the systems’ true dynamics. We hope to provide readers with potential insights and tools to overcome the challenging issues related to binding affinity prediction via docking.

## 1. Introduction

Docking, originally introduced by Kuntz et al. [1], is a computational method that virtually tries to predict a complex of (usually) two binding partners. Typically, these binding partners are biological macromolecules (e.g., protein, DNA/RNA, peptide) or small molecules (e.g., endogenous ligands, drugs). Although nowadays specific docking methods are available for distinct binding partners, such as HADDOCK for protein-protein docking [2], here we focus on the more traditional small-molecule molecular docking methods, such as GOLD [3,4,5], Surflex-Dock [6], AutoDock [7] and Glide [8,9,10], that are regularly utilized in structure-based drug design to predict ligand interactions with the target protein. In structure-based small-molecule docking a small ligand molecule is aligned inside the binding cavity of the target protein and the resulting docking pose is evaluated by a specific scoring function. The scoring function generates a score for each pose, and the resulting values are used to rank the different poses and ligands. In a methodological sense, there are two independent stages in the docking process: the pose generation and the scoring. The first refers to the methods which are used to create different ligand and protein conformations and aligning different ligand conformations within the binding site of the protein. The latter, the scoring, is required in the docking process for a quantitative estimation of the pose quality. As docking is typically utilized to screen extensive small-molecule (up to millions) chemical libraries, the pose generation and the pose quality evaluation must be carried out by fast methods i.e., the computational cost should be low. To fulfill this, several simplifications are needed in the overall docking process.

The first simplification in docking is related to water, as this solvent is neglected by most docking programs. Only very recently, have several docking methods been introduced where individual water molecules are included in the pose generation and evaluation phase [10,11]. The challenge in water description is related to the fact that these abundant molecules are fast moving and rotating and they participate in hydrogen bonding (H-bonding) as a donor and acceptor. This means that a change in the orientation of a single water molecule in the binding site not only has an effect on the neighboring waters, but also extends to the surrounding multiple hydration layers, thereby affecting the whole water network. In addition, the differentiation of strong and weak H-bonds in these interactions should be considered. Thus, the abundant possibilities in the water arrangement prohibit a feasible, explicit evaluation of all the potential water interactions. The current state of how the water can be treated explicitly in docking is reviewed in [12].

The lack of motion is another simplification in docking. However, the dynamic nature of the whole system in terms of entropy and enthalpy should be acknowledged. Whereas the ligand flexibility is typically included in the docking process, the same does not hold true for protein. Usually, protein is considered as rigid, with the exception of the rotating hydroxyl groups of serine, threonine and tyrosine residues. Obviously, these simplifications affect the quality of the generated poses, which may be artificial [13]. As a result, different approaches that consider the protein flexibility have been developed, such as ensemble docking [14], where docking is conducted in an ensemble of different protein conformations.

The third simplification in docking is related to the analysis of the interactions between the protein and the ligand. The different types of protein–ligand interactions (for non-covalent binders) include ionic interactions, hydrogen bonds and van der Waals interactions (including dispersion, polar and induced interactions). The most accurate way to estimate these interactions is with a quantum mechanics (QM) based approach [15]. However, in most cases QM methods are computationally too expensive for docking purposes. To speed up the interaction analysis, calculations are typically conducted with simple potential energy functions, usually related to force-fields or statistical potentials. While the current force fields and scoring functions are well parametrized, polarization effects and a detailed proton affinity estimation are still lacking.

Docking programs produce one (or several) different poses for every ligand, and further rank different compounds based on their scoring functions. A comparison of different docking programs is difficult as the data sets to estimate the docking performance are often of low quality and there is no consensus on which metrics to apply in these comparisons. For instance, binding affinities predicted by the docking might be incorrect, despite the correctly predicted binding pose. Another example is the case in which a particular docking method performs reasonably with one protein but with another protein, docking poses are constantly mispredicted. These problems are well explained in the work of Cheng et al. [16], in which the frequently used CASF-2007 data set was employed to evaluate docking performance. In the same work, evaluation problems were solved by using three different metrics, namely “docking power”, “ranking power” and “scoring power”. Recently Li et al. [17], described a fourth metric, called “screening power”. “Docking power” is the power to identify the native docking pose among the decoy poses, while “scoring power” is the ability to predict the binding affinity. In virtual screening campaigns, employment of “ranking power” is usually more appropriate, as it is the ability to correctly rank compounds according to their binding affinity. Also, the “screening power” is highly relevant for virtual screening, as it measures how well the method is able to identify the true binders from a random pool of ligands, including non-binders.

Docking is utilized as a tool in both virtual screening and compound optimization. There are several very comprehensive reports indicating unreliable binding affinity predictions by docking; a good summary of those studies has been published by Pagadala et al. [18]. Additionally, in order to achieve reliable binding affinity predictions, the old docking methods being updated and new methods have been published. New parametrizations and methods are based on ever increasing datasets and increased computational capacities, such as the implementation of QM in docking [19] and moving towards dynamic docking [20]. In our laboratory, we have carried out different virtual screening and docking experiments since the early 2000s. While we used docking as a stand-alone approach in our early studies, nowadays, to increase the quality of our results we are increasingly employing diverse methods in parallel to docking. In the following, we briefly discuss those theoretical aspects which have directed us to use docking in combination with other methods. First, we focus on the pose generation in docking and then, we provide a short overview of scoring function caveats. Finally, we discuss the role of water and (the lack of) dynamics in the docking process.

## 2. Pose Generation and Scoring Functions in Docking

### 2.1. Pose Generation

Ligand and protein conformational freedom is a huge challenge in docking. Widely used docking programs handle ligands as fully flexible, thus typically generating a very large number of conformations during the docking process. In addition to conformation generation methods, the quality of the docking will depend on the force field. It is evident that the conformational aspect of the process is well optimized as all the widely used docking methods are able to identify the correct bioactive conformation of a ligand (i.e., to recreate the X-ray pose) in several instances. For example, this was shown by Li et al. [17], Warren et al. [21], and the same conclusions were reached in the CASP2 competition [22]. However, these results do not necessarily imply that the pose generation produces the correct ligand conformation and binding pose in all instances. Based on these observations, it is apparent that the focus should be placed on the scoring function to increase the quality of docking.

### 2.2. Scoring Functions

The strength of a protein-ligand complex is related to the intermolecular interactions between these binding partners, solvent effects and dynamics. The most conservative method to estimate all of these simultaneously, is to apply all-atom molecular dynamics (MD) simulations. However, in order to avoid the significant computational costs related to these simulations, molecular docking utilizes scoring functions to provide a fast and crude estimation of the binding affinity. There are three main types of scoring functions: force-field based, knowledge-based statistical functions, and empirical scoring functions [23]. Force-field based methods utilize molecular mechanics functions for evaluating the direct interactions between a ligand and the protein, and solvent effects are typically evaluated by a generalized Born/surface area (GB/SA) type of approach [24], which is often based on the work of Wesson and Eisenberg [25]. Knowledge-based methods rely on statistical information derived from the existing ligand-receptors complex structures [26] in the form of distance-dependent atom-pair potentials. The third approach, empirical scoring functions [8] is based on the idea that all the relevant factors affecting the binding are expressed in the form of (preferably simple) equations, like those describing H-bonding, rotational/translational degrees of freedom and polar/lipophilic effects. In addition, these equations are balanced by using a regression-type approach; in the literature this approach is sometimes referred to as regression-based scoring.

#### 2.2.1. Enthalpy and Entropy

Scoring functions attempts to estimate the binding affinity, which is directly related to the Gibbs energy of binding. There are several ways to describe the partitions of binding energy and one of these is described in Equation (1). This partition by Ajay and Murcko [27], describes the binding energy as individual components: the solvation/desolvation energy (∆*G_solvent_*); the change in energy of the receptor and ligand due to complex formation (∆*G_conf_*); the change in energy due to specific interactions between the ligand and the receptor (∆*G_int_*); and the contribution due to changes in movement (rotational, translational, vibrational) (∆*G_motion_*).

(1) $$∆G_{bind}=∆G_{solvent}+∆G_{conf}+∆G_{int}+∆G_{motion}\;$$

What can we conclude based on Equation (1)? First, one must note that it is inadequate to only study the protein-ligand interactions. Additionally, it is important to understand how both interact with water before the formation of the complex and how water mediates this process. Also, one must recognize the fact that binding energy includes the conformational aspects of ligand and protein, and also changes in motion (this is mainly an entropic effect). As entropy is directly related to the motion and the temperature, a single protein-ligand complex pose may not provide enough information to reliably predict binding affinity.

Finally, how reliable is the estimation of the strength of the direct interactions (∆*G_int_*) between different binding partners (protein-ligand-solvent)? This depends greatly on the description of the ionic interactions and van der Waals interactions (including H-bonding). The entropic component is thought to be related mainly to the conformational and rotational/translational aspects, but we believe this is an optimistic view. More emphasis should be placed on how much the protein flexibility contributes to the stability of the protein-ligand complex and how the water affects the binding energy.

#### 2.2.2. Direct Interactions

Direct polar interactions between ligand, protein and water are enthalpic in nature. In scoring functions, these interactions are considered by specific terms such as H-bonding, Lennard-Jones type of functions and ionic interactions. Dispersion-type interactions (erroneously called van der Waals interaction) are usually reasonably described by classical Lennard-Jones potential. As indirect proof of how precise these equations are, even the latest parametrization of the OPLS (Optimized Potentials for Liquid Simulations) force field family, OPLS3 [28], utilizes the formerly developed Lennard-Jones parameters. Indeed, many of the scoring functions use this approach to model dispersion [5,24,29], although GOLD uses softer 8–4 potential while Dock and Glide prefer 12–6 potential [1,3,4,5,8,9,10]. Other approaches do exist, for example Surflex uses surface-based description (derived from van der Waals surface) [6] and FlexX has a scoring term based on separate attractive terms for H-bonds, ionic, aromatic and lipophilic interactions and atom-center distance-based repulsive function [30].

One could argue that a proper description of dispersion is needed for accurate ligand-binding prediction, however, a precise understanding of the H-bonding is even more important. Recently, Raschka et al. analyzed the type of interactions found in 136 non-homologous protein-ligand complexes [31], concluding that strong H-bonds are required for most high-affinity ligands. In addition, they disclosed that the protein prefers to act as a H-bonding donor. As an explanation of why the protein prefers to act as a H-bond donor for high-affinity ligands, the authors speculated that geometrically more constrained H-bonding donors were enriched during the evolution. Consequently, a proper H-bonding description in scoring functions is required.

In all scoring functions, both distance and angular parameters are included in the H-bond potentials in similar fashion, in several force fields. In addition, the type of H-bonding (e.g., charged, neutral) is considered. One of the most detailed forms of H-bonding potential is implemented within the Glide XP [10], where three different types of H-bond are used: neutral-neutral, neutral-charged and charged-charged. The functional form of the Glide XP includes several H-bond class-specific modifications and environment-based restrictions. As a result, enhanced recognition of the “false positive” H-bonds is achieved.

#### 2.2.3. Hydrogen Bond Strength and Classification

The hydrogen-bond (H-bond) is mainly an electrostatic interaction, which is typically modelled via Coulomb-type-equations, and for this, the dielectric constant is a critical factor. Unfortunately, a reliable and fast method to calculate the dielectric constant does not exist. One approach is to use QM/MD-methods but this approach is currently too slow for docking purposes [15]. Furthermore, the challenge in H-bond modeling is the high variability of different H-bond types and strengths. Even the environment has a huge effect as demonstrated with water–water H-bonds [32]. In a neutral (pH 7) environment a single water-water H-bond is weak but in basic or acidic medium it becomes a 6-fold stronger and 15% shorter charge assisted H-bond. In fact, H-bond strengths can usually be estimated based on the proton affinities or pK_a_-values of both the donor and acceptor site. Based on this approach, a 6-class classification for H-bonding has been developed [33]. Normal, weak H-bonds (those without charge) are the most common type of H-bonds found in biological systems. These are unassisted by charge or resonance and thus are weak, asymmetric and driven by electrostatic force. On the other hand, all the H-bonds assisted by charge (either negative or positive or both) are strong and short, if the pK_a_-values of donor and acceptor match. When pK_a_-values mismatch (>2 pK_a_ units), these H-bonds are classified as regular H-bonds. In a biological system this means that the ionization state of the protein and ligand are needed for proper H-bonding evaluation, and also the pK_a_-values are required. Most of the current H-bond potentials produce reliable predictions for uncharged H-bonds. The same does not hold true for the H-bonds that include ionizable donor and acceptor groups. There are several computational methods available for both protein and ligand pK_a_-value calculations [34,35,36]. Nevertheless, proton affinities are hardly ever considered in scoring functions. Therefore, the Glide approach [10] of differently scoring H-bonds with charge, is probably correct.

## 3. Water, Dynamics and Docking

Water has an important role in the biological environment, especially in the protein matrix [37,38]. The crucial role of the water in the ligand binding process has long been acknowledged [39]. Water has an important role in ligand binding thermodynamics [40,41], even in the environment of a lipophilic binding cavity [42] and displacing specific water molecules from the binding site may play an important role in the ligand optimization process [43]. Moreover, water related H-bonding networks have a significant influence in the structure-activity relationship [44], and optimizing the ligand taking into account the surrounding water network may result in enhanced binding affinity and prolonged residence time [45]. The problem is that detailed information of how water is located within and around the ligand binding site is mostly unavailable. The most common tool for determining 3D structure, X-ray crystallography, can only provide partial information because the resolution and low-quality electron density limits water detection. Those water molecules which are detected by X-ray are often entropically stabilized [46]. In addition, crystallization conditions are typically far from the biologically relevant ones, and also the co-crystallized ligand molecule(s) may influence the observed hydration network (differently when compared to a docked ligand).

Easy application of water placement in docking is restricted because the water in the binding site is heterogenous. In different locations, an individual water molecule has restricted rotational freedom and H-bonding capabilities. The terminology, “happy” and “unhappy” water has been introduced to describe the individual water energies compared to bulk water [47]. Happy and unhappy water refer to low-energy and high-energy water, respectively. The unhappy water molecules within the binding site have either lost their degree of freedom (entropic penalty) or they are incapable of fulfilling all possible H-bonds (enthalpic penalty), which result in higher energies compared to the bulk water. Therefore, displacing unhappy water molecules from the binding site with the ligand results in a gain in binding affinity [48]. On the other hand, displacing a happy water molecule from the site is typically unfavorable. Furthermore, not all regions within the binding sites are hydrated and occupied by water molecules [49,50]. Areas exist that are energetically so unfavorable for water to occupy that there is no water present; instead, they appear as dry void regions (also referred to as vacuum or dewetted regions) [51,52]. Occupying these regions with a ligand molecule results in both more favorable enthalpy and entropy of binding. The reason for this gain in binding affinity is the fact that the increased protein-ligand interaction surface results in stronger van der Waals interaction. In addition, filling the dewetted region increases entropy. In accordance with this, we have noticed that these vacuum sites played a significant role in determining the compound activity in our series of Autotaxin inhibitors [53].

Even though water has been acknowledged to play an important role in binding, de novo placement of water has not been explicitly included in docking methods, with the exception of Glide XP [10]. The Glide XP includes terms for the hydrophobic enclosure, which promotes the insertion of the lipophilic parts of the ligand in the protein’s lipophilic cavities; thereby, simulating the displacement of potential high-energy water. Moreover, in this method, by utilizing a grid-based methodology “virtual waters” are placed into the binding site, and penalties for are given for improperly solvated hydrophilic (polar or charged) groups and for the water that makes an unusual number of hydrophobic contacts.

As already stated, docking is usually unable to provide a good estimate of the role of the solvation penalties related to the binding. As a result, several complementary computational methods have been developed to identify and analyze water molecules around the protein-ligand complex to estimate its role in binding. Different approaches have been reported and the most popular methods are reviewed by Bodnarchuk [54]. For instance, the Schrödinger’s WaterMap uses a short MD simulation and the estimation of the energies of the hydration sites are derived based on the simulation [48,50], whereas in the Molecular Operating Environment (MOE), the binding desolvation penalties can be estimated by 3D reference interaction site model (3D-RISM), which is based on the density functional theory of liquids [55,56]. The main limitation of these methods is that they are heavily dependent on the protein conformation used in the calculation. To exemplify this, a parallel calculation of the hydration site energy with the same protein may produce totally different results, even if only minor protein conformational change occurs or only one side of the chain conformation is altered. This limitation should be kept in mind when utilizing these methods, as for example, a conformational “induced-fit” effect upon ligand binding (via docking) might hamper the results [57]. Although these methods are now becoming increasingly popular and have demonstrated usefulness in explaining lead molecule structure–activity relationships [58], it is still unclear if these methods are applicable in virtual screening campaigns. One of the first attempts to include these computational approaches directly into scoring functions is WScore [11]. In WScore, a default WaterMap calculation with the apo-protein is utilized to gain insight into the hydration site positions and their corresponding energies. The occupancy of these hydration sites by a ligand are included in the scoring. Moreover, an ensemble docking is carried out that aims to take into account the protein flexibility, which as mentioned above, is the major issue with the WaterMap. The usefulness of WScore and other related methods remain to be seen. Furthermore, conventional MD simulations can be applied to evaluate the hydration networks; thus, some errors related to force field accuracy may arise [59]. In a way, we agree with the statement by Hummer [60], that the contribution of water for the ligand binding may be substantial but its evaluation is challenging.

One of the shortcomings of docking is that it produces only a snapshot of the putative binding conformation. This is a notable limitation, as in real-life the binding event is not a static event, it is dynamic. For instance, we observed a good example of this in a study of 1-/2-monoacylglycerol hydrolysis by Monoacylglycerol lipase (MAGL) [61]. Whereas the wild-type MAGL hydrolyzes both substrates at an identical rate, a C242A mutation in the active site impairs the hydrolysis of the 1-acylglycerol but not the 2-acylglycerol. This mutation had no effect on the binding conformations obtained by the docking; but, it was unable to provide an explanation for the observed difference in the hydrolysis among the substrates. However, in this case even short MD-simulations were capable of highlighting the differences in the substrate binding dynamics that arose due to the mutation.

Perhaps due to the fact that docking is currently unable to consider the impact of water and the dynamic nature of binding, applying MD simulations for the docking pose validation has attracted growing interest in the scientific community [62,63,64,65,66,67]. This is probably also due to increased accessibility to adequate computing resources (e.g., GPUs) that are required for simulations with a reasonable time-scale. Another factor that has made MD simulations more relevant is the improvement in the force fields that are now capable of handling both small molecules and proteins with reasonable accuracy. These improvements have led to more relevant observations from the simulations. Interestingly, MD simulations appear to provide the solution to the two issues that docking is incapable of handling—water and the dynamics.

## 4. Solution

In this opinion paper, we have exemplified the underlying issues in predicting binding affinity via docking. The main issues are related to the H-bonding and the water description, and how water and the protein-ligand complex should be considered as a dynamic system. While describing the H-bond is clearly an issue, we should also acknowledge that this has already been quite well described in modern force fields. For example, the new OPLS3 and recent AMBER (Assisted Model Building with Energy Refinement) and CHARMM (Chemistry at Harvard Macromolecular Mechanics) force fields include a better H-bonding description [28,68,69]. Additionally, MD is becoming an increasingly robust method to study individual protein-ligand complexes. Unfortunately, the computational costs of MD are still too high to allow virtual screening.

What can be done to increase the accuracy of the binding affinity prediction? With current methods, resolving this issue is extremely challenging. For H-bonding, it is feasible to include a more precise energy evaluation method that would allow recognition and differentiation of the strong and weak H-bonds. However, this requires a fast and reliable pK_a_-value calculation that also considers conformational and environmental aspects of the binding cavity. Furthermore, due to the active role of the water in binding, it is obvious that water needs to be explicitly included in the docking process. All the current evidence contradicts docking in the gas phase. WaterMap and other related methods have partially resolved this issue but a more comprehensive solution is required. Finally, implementation of dynamics in scoring functions remains challenging. In future, scoring functions need to be reinvented so that they are able to describe the dynamics related to the binding. Overall, new approaches are required to address the issues discussed above.

Our current solution is based on two comprehensive approaches, one to use docking tools in more efficient ways [53,70], and the other is to use MD simulations to validate the results of the classical docking [71,72]. Prior to any docking experiment, one should explore the flexibility of the target protein, based on both the existing protein structures and MD simulations. At the same time, it is of utmost importance to determine the solvation status of the binding cavity and the energy levels of the potentially happy and unhappy water. Subsequently, this information is further applied in docking by utilizing suitable constraints. This approach can help us to identify more reliable binding poses. Finally, the most promising poses are further analyzed by short (usually 200 ns) MD simulations and followed by WaterMap analysis. However, our approach has two major shortcomings: It is slow and difficult to implement. These shortcomings are tolerable, as long as we have sufficient computing resources and an adequate amount of time to work with the target. We resolve the H-bond issue by estimating the pK_a_-values with different computational methods (e.g., QM-polarized docking). Lastly, even after implementing all of these user-based interventions, we always use the most sophisticated scoring function, the eye. If you trust your docking pose, you might be right.
